# Efflux transporters in blood-brain interfaces of the developing brain

**DOI:** 10.3389/fnins.2015.00021

**Published:** 2015-02-05

**Authors:** Nathalie Strazielle, Jean-François Ghersi-Egea

**Affiliations:** ^1^Brain-iLyon, France; ^2^Oncoflam Team, Lyon Neuroscience Research Center, INSERM, U1028, CNRS, UMR5292, Université Lyon 1Lyon, France; ^3^BIP Platform, Lyon Neuroscience Research Center, INSERM, U1028, CNRS, UMR5292, Université Lyon 1Lyon, France

**Keywords:** blood-brain barrier, choroid plexus, development, ABC transporter, SLC transporter, perinatal injury, hypoxia, xenobiotic

## Abstract

The cerebral microvessel endothelium forming the blood-brain barrier (BBB) and the epithelium of the choroid plexuses forming the blood-CSF barrier (BCSFB) operate as gatekeepers for the central nervous system. Exposure of the vulnerable developing brain to chemical insults can have dramatic consequences for brain maturation and lead to life-long neurological diseases. The ability of blood-brain interfaces to efficiently protect the immature brain is therefore an important pathophysiological issue. This is also key to our understanding of drug entry into the brain of neonatal and pediatric patients. Non-specific paracellular diffusion through barriers is restricted early during development, but other neuroprotective properties of these interfaces differ between the developing and adult brains. This review focuses on the developmental expression and function of various classes of efflux transporters. These include the multispecific transporters of the ATP-binding cassette transporter families ABCB, ABCC, ABCG, the organic anion and cation transporters of the solute carrier families SLC21/SLCO and SLC22, and the peptide transporters of the SLC15 family. These transporters play a key role in preventing brain entry of blood-borne molecules such as drugs, environmental toxicants, and endogenous metabolites, or else in increasing the clearance of potentially harmful organic ions from the brain. The limited data available for laboratory animals and human highlight transporter-specific developmental patterns of expression and function, which differ between blood-brain interfaces. The BCSFB achieves an adult phenotype earlier than BBB. Efflux transporters at the BBB appear to be regulated by various factors subsequently secreted by neural progenitors and astrocytes during development. Their expression is also modulated by oxidative stress, inflammation, and exposure to xenobiotic inducers. A better understanding of these regulatory pathways during development, in particular the signaling pathways triggered by oxidative stress and xenobiotics, may open new opportunities to therapeutic manipulation in view to improve or restore neuroprotective functions of the blood-brain interfaces in the context of perinatal injuries.

## Introduction

Blood-brain interfaces comprise the cerebral microvessel endothelium forming the blood-brain barrier (BBB) and the epithelium of the choroid plexuses forming the blood-CSF barrier (BCSFB). These interfaces operate as gatekeepers for the central nervous system, hence providing the cerebral homeostasis necessary for normal neuronal growth and activity.

During brain development, these CNS barriers display stage specific properties. For instance, in rat, the activity of gamma-glutamyl-transferase, an enzymatic marker of brain microvessels, continuously increases from birth to adulthood (Betz and Goldstein, 1981; Gazzin et al., 2008). Conversely, the metabolic barrier to DOPA mediated by DOPA-decarboxylase is most effective in young animals (Betz and Goldstein, 1981). Blood to brain influx of pyruvate, lactate and β-hydroxybutyrate, as well as large neutral amino acids is greater in suckling rats compared to adult animals, while active efflux of amino acids across the BBB may not occur before 21 days of age (review in Betz and Goldstein, 1981). In the choroid plexus epithelium, the composition in tight junction proteins changes during brain development. Notably, expression of the pore-forming claudin-2 is upregulated and that of the tightening claudin-3 is downregulated, probably reflecting perinatal changes in inorganic anion transport and related CSF secretion rate at the BCSFB (Kratzer et al., 2012). These various examples illustrate changes in barrier functions that occur to face specific needs of the brain at different developmental stages.

The vulnerability of the developing brain to chemical insults, especially during the late prenatal/early postnatal period (herein referred to as perinatal period) can have dramatic consequences for brain maturation, leading to life-long neurological diseases (Kaindl et al., 2009; Landrigan and Goldman, 2011; Miodovnik, 2011). The ability of blood-brain interfaces to efficiently protect the immature brain is therefore a major pathophysiological issue. It is also key to our understanding of therapeutic drug entry into the brain of neonate and pediatric patients. Little is known about the mechanisms protecting the developing brain from harmful compounds and regulating the cerebral entry of pharmacologically active compounds. The cerebral interstitial fluid concentration of xenobiotics is set by their ability to cross the brain endothelium and the choroidal epithelium, as well as a number of other parameters. These include protein binding, local cerebral capillary density and cerebral blood flow, intraparenchymal diffusion rate, movement by fluid flow along perivascular spaces and within ventricular and cisternal spaces, and CSF turnover (reviewed in Ghersi-Egea et al., 2009; Westerhout et al., 2011). These parameters change during development. Plasma protein concentration is lower during development than in adult, so are cerebral blood flow and capillary density (Caley and Maxwell, 1970; Nehlig et al., 1989). Intracerebral fluid dynamic differs between adult and developing brain. CSF turnover increases at birth, and the total CSF (cisternal, subarachnoid, and ventricular) to brain volume ratio is largely higher in perinatal brain (unpublished data). Taken together, these observations indicate that the impact of blood-CSF exchanges on the overall cerebral bioavailability of drugs or toxic compounds is likely to be more important in young than in adult individuals. At the level of the BBBs proper, the neuroprotective functions result from intercellular tight junctions sealing the barrier cell layers, as well as detoxification enzymes and efflux transporters located in the barrier cells (Strazielle and Ghersi-Egea, 2013). It is now clearly established that non-specific paracellular diffusion through the cerebral endothelium and the choroidal epithelium is efficiently restricted by the tight junctions early during development (Liddelow et al., 2013).

**Figure 1:**
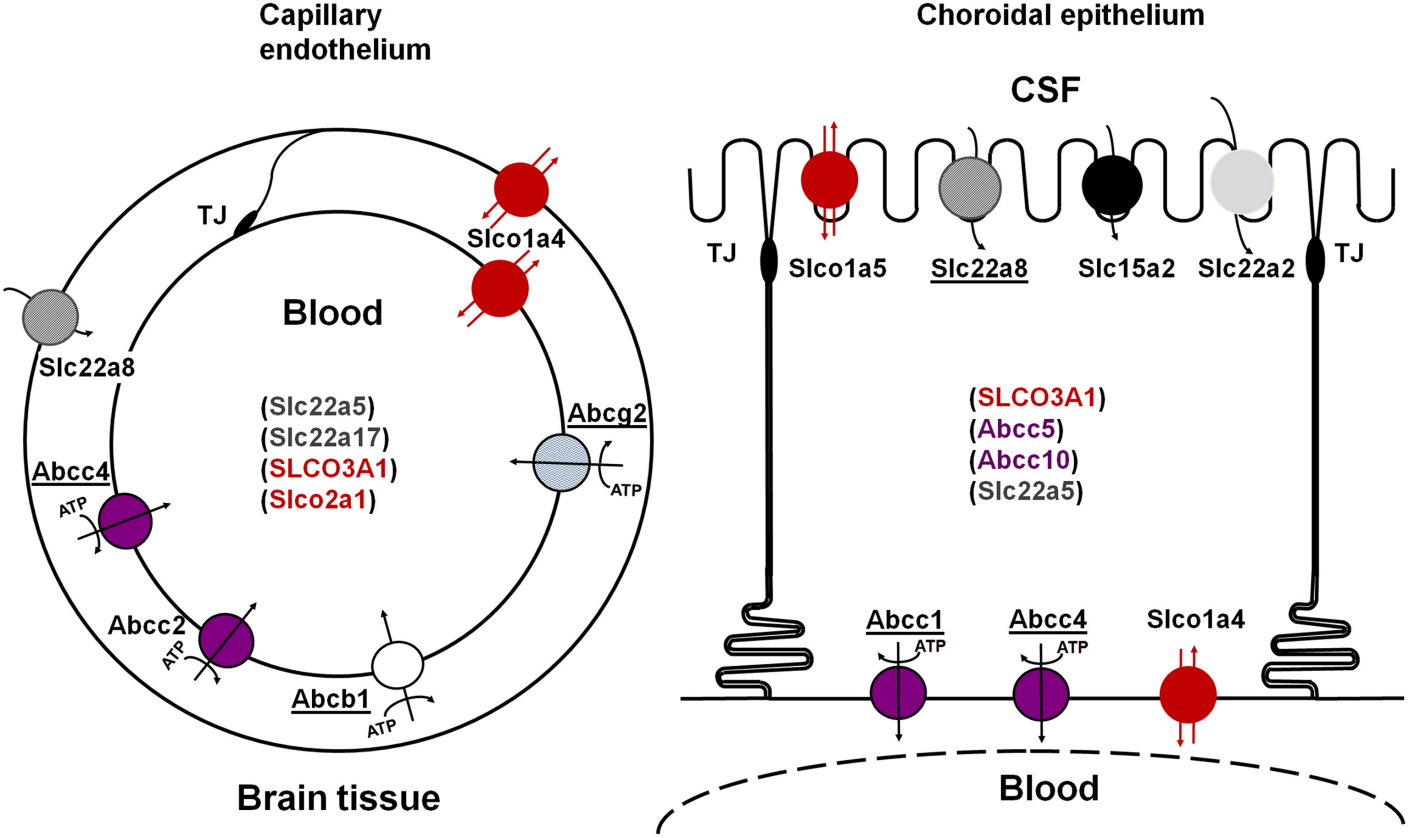
**Schematic representation of the main multispecific transporters involved in neuroprotective efflux at the blood-brain (left) and blood-CSF (right) barriers**. Underlined names represent transporters for which evidence has also been reported in human. The ATP-dependency is shown only for primary ATP-dependent transporters. Transporters in parenthesis are carriers for which evidence of their involvement in CNS-blood efflux processes is still limited. See text for references. TJ, tight junction.

This review focuses on efflux transporters and analyses the data relative to their developmental pattern of localization, expression and function. These multispecific transport systems play a key role in preventing the entry into the brain and CSF of numerous blood-borne xenobiotics, including drugs, or in increasing the clearance of potentially harmful organic ions from the brain. They include ATP-Binding Cassette (ABC) transporters of the ABCB, ABCC, and ABCG families, solute carriers for organic anions and cations of the SLC21/SLCO and SLC22 families, and peptide transporters of the SLC15 family ^1^ (Figure 1). These efflux transporters also recognize biologically active endogenous compounds, including steroid hormones and eicosanoids (leukotrienes, prostaglandins), thus protecting the brain from a potentially deleterious overproduction of these compounds.

### ATP-binding cassette transporters

Multispecific efflux transporters that belong to the ABC transporter superfamily and are found in blood-brain interfaces include ABCB1 (P-glycoprotein), ABCG2 (breast cancer resistance protein, Bcrp), and several ABCCs (multidrug-related resistance proteins, Mrp) (Schinkel et al., 1996; Nishino et al., 1999; Wijnholds et al., 2000; Cooray et al., 2002; Strazielle and Ghersi-Egea, 2013). Located at the blood-facing membranes they prevent brain penetration of their substrates by an energy-dependent, unidirectional, outwardly directed transport mechanism. They are responsible for the efflux of many structurally unrelated lipophilic and amphiphilic environmental toxic compounds or drugs, including anti-inflammatory, immunosuppressive, anti-infectious, and antineoplastic drugs, some antiepileptic, antidepressant and psychotropic agents, and drug conjugates in the case of ABCCs (Schinkel and Jonker, 2003; Leslie et al., 2005; Staud and Pavek, 2005; Strazielle and Ghersi-Egea, 2005). These ABC transporters are differentially localized between the two main blood-brain interfaces (Figure 1). ABCB1 and ABCG2 are highly expressed at the BBB, while ABCC1 is a landmark of the BCSFB (Daood et al., 2008; Gazzin et al., 2008). Abcc2 has been described in the rodent BBB (e.g., Wang et al., 2014), and Abcc4 expression and function have been described in both interfaces in mice (Leggas et al., 2004). Species differences are found, namely for ABCG2 which is likely to play a more important protective function in human than in rodent (Shawahna et al., 2011), and ABCB1, which is encoded by a single gene in human and 2 genes in rat and mouse. Species difference may also exist for ABCC2 whose expression at the BBB in normal human brain is still an open question (Kubota et al., 2006; Dauchy et al., 2008).

Several studies suggest that ABC transporters are already functional at the blood-brain interfaces in neonates, but that their expression is developmentally regulated in a protein- and barrier-specific manner.

#### Developmental pattern of ABCB localization, expression and activity at blood-brain interfaces

In mouse and rat, the Abcb1-dependent phenotype of multidrug resistance is associated with 2 genes Abcb1a and Abcb1b, both homologs to the human ABCB1 transporter gene. Abcb1a and Abcb1b are expressed in a tissue-specific manner, and also differ to some extent in their transport properties. Abcb1a is closer to the human transporter in terms of amino acid primary sequence and gene regulatory region, and is the predominant gene expressed at the BBB. Its expression is already detectable in endothelial cells in the neural tube of 10.5-day-old mouse embryos (Qin and Sato, 1995). Immunohistochemical analysis performed with different antibodies on human tissues showed that Abcb1 protein consistently localizes at the membrane of brain microvessels by mid-gestation. Before this stage, the signal is heterogeneous and displays regional differences (Schumacher and Mollgard, 1997; Virgintino et al., 2008). Accordingly, Gazzin and coll. reported that this transporter is present in rat brain microvessels at birth, a developmental stage which corresponds to preterm development in human (Gazzin et al., 2008). As would be required for its neuroprotective efflux function, Abcb1 immunoreactivity was found at the luminal membrane of the endothelial wall. Because the cerebral capillary density increases greatly during development, the amount of Abcb1 protein at the BBB cannot be compared between stages using total brain homogenates. A standard curve-based quantitative Western blot analysis performed on isolated microvessels showed that the amount of Abcb1 in the BBB in 9- and 17-day-old rats was only one-fifth of that found in microvessels isolated from adult animals (Gazzin et al., 2008, 2011). This is in agreement with the more intense immunohistochemical signal observed in brain microvessels from adult as compared to perinatal or postnatal developmental stages in human, rat or mouse (Matsuoka et al., 1999; Tsai et al., 2002; Daood et al., 2008; Gazzin et al., 2011). It is also consistent with the 3-to-4-fold increase in Abcb1a mRNA levels measured in endothelial cells from adult mice or rats compared to levels in endothelial cells of developing animals (2- to 8-day-old mouse, 14-day-old rat) (Daneman et al., 2010a; Harati et al., 2012a). The low expression and protein level of Abcb1 observed at the BBB in different species at late prenatal and early postnatal developmental stages may therefore result in a weaker protection of the brain against circulating compounds that are handled by this transporter. Accordingly, a 80–90% lower brain accumulation of cyclosporin A and digoxin, assessed as brain-blood/plasma ratio 2 h following administration, was observed in adult mice compared with 1-day-old mice, a difference which was abolished in Abcb1a deficient animals (Goralski et al., 2006). A number of other functional studies have been conducted using digoxine as Abcb1 substrate. A greater accumulation of this molecule was observed in the brain of 15.5-day-old embryonic mice compared to the brain of 18.5-day-old embryonic mice (Petropoulos et al., 2010). In rat, the unbound brain-to-plasma concentration ratio of the molecule measured at steady-state was not different between 3-week- and 12-week-old animals (Harati et al., 2012a). A positron emission tomography study conducted in rhesus monkeys showed a significantly higher clearance value for verapamil uptake in 9-month-old animals than in 2- and 4-year-old animals (Takashima et al., 2011). With the reservation that substrate specificities for Abcb1 are not absolute, in particular for digoxin which can be recognized by other transporters such as Slco1a4/oatp2, overall, these partial data suggest that ABCB1 functional activity parallels gene expression levels, both increasing from prenatal to pre-adult stages in various animal species, including non-human primates.

The amount of ABCB1 protein in rat and human choroid plexus is low by comparison to the levels measured in brain microvessels (Gazzin et al., 2008), and its cellular and subcellular localization within the choroidal tissue is subject to conflictual results. Its implication in the regulation of exchanges across the BCSFB is likely limited. In contrast to the BBB, Abcb1a mRNAs are not detected by RT-PCR in the BCSFB. The gene expressed in rat choroid plexus is Abcb1b. The level of expression, very low in adult rat tissue, is 4–8 times higher during the perinatal period of development, with a peak on postnatal day 2 (Kratzer et al., 2013). The actual functional activity of the transporter and the significance of this perinatal upregulation of choroidal Abcb1b expression are not known.

#### Developmental pattern of ABCG2 localization, expression and activity at blood-brain interfaces

There are few data available on the developmental expression of ABCG2, but there is a general agreement that the transporter is associated with the brain vasculature and localizes at the luminal membrane of brain endothelium early during development, specifically by week 22 of gestation in human (Daood et al., 2008), embryonic day 12.5 in mouse and in rat (Tachikawa et al., 2005; Orford et al., 2009; Ek et al., 2010). No apparent difference in signal intensity was observed throughout development in these studies. Only a moderate (2-fold or less) increase in Abcg2 mRNA levels was observed in endothelial cells isolated from adult vs. postnatal mouse brain (Daneman et al., 2010a) or between cerebral capillary preparations of comparable purity isolated from 14-day-old rats vs. adult animals (Harati et al., 2012a). In the latter study no difference in Abcg2 protein level was observed by Western blot between the two stages. Functional studies on Abcg2 mediated transport during development have been performed only in rat on postnatal day 21. At that stage, blood-to-brain prazosin transport was not different from that measured in adult rats (Harati et al., 2012a). Overall, ABCG2 seems to reach high levels of expression at the BBB early during development. It may compensate for the lack of Abcb1-dependent protection at a period of high brain vulnerability.

Studies on the expression and function of Abcg2 in choroid plexus generated apparently conflicting results, possibly linked to species differences, or due to difficulties in achieving specific immunolabeling in this tissue. The level of protein assessed by Western blot is clearly much lower in choroidal tissue than in brain capillaries, as illustrated in the work performed by Reichel and colleagues in rat (Reichel et al., 2011). They loaded 10 times more proteins from choroidal tissue than from microvessels to be able to detect Abcg2 in the former sample, while the capillary fraction yielded an intense signal. In line with that report, an immunohistochemical study conducted on human tissue failed to detect ABCG2 in the choroid plexus at any developmental stage (extending from 22 weeks of gestation to adult), while the transporter was clearly detected in the microvasculature (Daood et al., 2008). Using transgenic mice expressing GFP under the control of Abcg2 promoter/enhancer sequences, Orford and colleagues showed that the transporter is associated with choroidal vessels from early on during development but not with the choroidal epithelium (Orford et al., 2009). By contrast, the protein was detected at the apical membrane of the choroidal epithelium in adult mouse, a signal that was not seen in Abcg2 knock-out animals (Tachikawa et al., 2005; Zhuang et al., 2006). No developmental data was provided in these studies. Other studies failed to detect the protein in choroid plexus epithelium of the adult rat (Roberts et al., 2008; Ek et al., 2010). One of the latter studies reported Abcg2 immunoreactivity at the basolateral membrane of the choroidal epithelium only at early developmental stages (embryonic day 15 and postnatal day 1) (Ek et al., 2010). This pattern of Abcg2 protein expression in rat parallels the temporal profile of choroidal Abcg2 mRNA levels which are also higher in pre- and perinatal stages than in adult (Ek et al., 2010; Kratzer et al., 2013). It has to be noted that mRNA levels are low in choroid plexus in both rat and mouse (Tachikawa et al., 2005; Kratzer et al., 2013). Overall, if Abcg2 has any significant transport function at the BCSFB, it should be especially relevant to the developing period.

#### Developmental localization, expression and activity of efflux transporters of the ABCC family at blood-brain interfaces

An immunohistochemical signal for ABCC1 was consistently observed in human choroid plexus epithelium early in gestation, from week 22 onwards (Daood et al., 2008). In rat, Abcc1 protein was detected in choroid plexus epithelial cells as early as embryonic day 15, with a basolateral localization consistent with its efflux transporter activity and a neuroprotective function (Gazzin et al., 2008; Ek et al., 2010). Choroidal mRNA levels were 2- to 3-fold lower in 15-day-old embryos and 7-day-old rat pups than in adult (Ek et al., 2010; Kratzer et al., 2013), but at birth, protein levels measured by quantitative Western blot are similar to those in adult (Gazzin et al., 2008), suggesting that Abcc1-mediated efflux at the BCSFB is active early during development. Protein and mRNA levels of Abcc1 are low in both the rodent and human microvasculature in comparison to choroidal levels, and the actual localization and efflux activity of the protein at the BBB proper remains controversial (reviewed in Gazzin et al., 2008). One study investigated ABCC1 protein in the human BBB during development and failed to detect the transporter at any developmental stage (Daood et al., 2008). Only rat mRNA expression data are available concerning the developmental pattern of other Abcc transporters. In the choroid plexus, the temporal expression profile for Abcc4, for which functional evidence of transport has been provided *in vivo* in adult mice (Leggas et al., 2004), follows a developmental pattern similar to that of Abcc1, with a limited increase in mRNA level in adult tissue compared to tissue from embryonic day 19. As in choroid plexus, mRNA levels in brain endothelial cells are moderately (3.3 times) lower in young mice (postnatal day 2–8) than in adult animals (Daneman et al., 2010a). The concurrent expression of this transporter in the BCSFB and the BBB from early stages on, and its polarized localization at blood-facing membranes in both barriers suggest that it plays an important role in the clearance of endogenous substrates in young and adult individuals.

Abcc5, Abcc9, and Abcc10 genes investigated in rat choroid plexus are expressed at a similar or higher level in developing animals compared to adults (Kratzer et al., 2013). Functionally, an Abcc-dependent basolateral efflux of intracellularly formed drug conjugates has been demonstrated in freshly isolated choroid plexuses or in choroid plexus epithelial cells, both obtained from 2-day-old rats (Strazielle and Ghersi-Egea, 1999; Ghersi-Egea et al., 2006). These *ex vivo*/*in vitro* studies indicated that one or several Abcc proteins are efficiently participating to a neuroprotective enzymatic BCSFB during development.

### Solute carrier proteins

The SLC superfamily comprises virtually all transporters that do not belong to the ABC family. Multispecific SLC transporters responsible for the neuroprotective efflux of exogenous and endogenous toxic molecules from brain or CSF to blood belong mainly to three subfamilies. The SLC22 subfamily includes organic anion and cation efflux transporters also referred to as OAT, OCT, and OCTN proteins. SLCO transporters, previously known as SLC21 form another subfamily of organic anion transport polypeptides (OATPs). Of note, several SLCO rodent genes have no homologs in human, and vice-versa. SLCO and SLC22 transporters accept as substrates a broad range of cationic and anionic compounds, including environmental pollutants, and various drugs such as antibiotics and nucleosidic antiviral drugs, non-steroidal anti-inflammatory agents, and some antiepileptic drugs. They also transport steroid or drug conjugates, and lipid mediators (Strazielle et al., 2003; Hagenbuch and Stieger, 2013; Koepsell, 2013). Finally, members of the SLC15 family, known as peptide/proton cotransporters, transport in addition to endogenous di- and tripeptides, some peptidomimetic drugs, e.g., β-lactam antibiotics, antiviral nucleoside prodrugs or angiotensin converting enzymes (Daniel and Rubio-Aliaga, 2003).

Some of these solute carriers are bidirectional. Others function as unidirectional inwardly-directed multispecific transport systems and exchange their substrates with specific intracellular divalent organic anions by a coupled secondary energy-dependent mechanism. SLC transporters that are involved in brain efflux localize at the abluminal (BBB) or apical (BCSFB) membrane of barrier cells, where they remove drugs and toxic compounds from brain extracellular and CSF spaces. The most documented SLC efflux transporters at blood-brain interfaces are the following. SLC22A8/OAT3 is present at both brain endothelial cells and choroidal epithelial cells in rodents, and is also detected in human choroid plexus (Nagata et al., 2002; Alebouyeh et al., 2003; Kikuchi et al., 2003; Mori et al., 2003; Ose et al., 2009). Slco1a5/Oatp3, Slc15a2/Pept2, and possibly Slc22a2/Oct2 are restricted to choroid plexus (Kusuhara et al., 2003; Ohtsuki et al., 2003). Other organic anion transporters are found at blood-brain interfaces, but their possible involvement in brain efflux rather than influx processes remaining to be established (Figure 1). Slco1a4/oatp2 is found at both luminal and abluminal membranes of brain endothelial cells, and only at the basolateral membrane of the choroidal epithelium in rat (Gao et al., 1999). In human, two SLCO3A1 variants v1 and v2, of similar and broad substrate specificity, are respectively located on the basolateral and apical membranes of the choroidal epithelium, suggesting that SLCO3A1 may function both as an influx and an efflux transporter (Huber et al., 2007). SLCO1A2 and SLCO2B1, also expressed at the human BBB, are likely to mediate brain influx rather than brain efflux, on the basis of their localization at the luminal membrane of the endothelium (Gao et al., 2000; Bronger et al., 2005).

#### Developmental localization, expression and activity of SLC22 and SLCO efflux transporters at blood-brain interfaces

Data on the developmental expression of efflux transporters that belong to the SLC22 and SLCO gene families are scarce and obtained only from rat and mouse studies. Transcriptomic analysis of endothelial cells isolated from adult vs. postnatal mouse brains suggests that developmental profiles are heterogeneous among genes (Daneman et al., 2010a). While mRNA levels for Slc22a5 (octn2) and Slco1a4 were 2-to 5-fold lower in endothelial cells from developing animals compared to adults, Slc22a8 expression was similar at both stages. Slc22a17 and Slco2b1, whose precise functions are currently unknown at brain barriers, did not display developmental changes either. Of note in this study, the mRNA levels of Slco2a1, the prostaglandin transporter with an unknown directionality of transport, were much higher at the BBB from developing vs. adult mice. This suggests a developmentally regulated mechanism for blood-brain exchange of prostaglandins, possibly related to the function of these lipid mediators in brain maturation. In rat, a lower expression was reported for Slco1a4 between developing and adult animal, while there was no change for Slc22a8 (Harati et al., 2012a). This was assessed by quantitative PCR and Western blot on isolated capillaries and confirms the mouse transcriptomic observations.

The analysis of transcriptomic profiles established in rat choroid plexus for 14 Slco and Slc22 members showed that most genes, including Slc22a5, Slco1a4, and Slco1a5 are expressed at similar or higher levels during the perinatal period than at the adult stage. One exception is Slc22a8, for which expression levels are respectively 4-fold and 2-fold lower in 19-day-old embryos and 2-day-old rats than in adults. At an earlier stage, in 15-day-old embryos, gene expression levels relative to adult were more heterogeneous (Kratzer et al., 2013). A functional analysis showed that *ex vivo* uptake of bromosulfophthalein, a typical Slco substrate, was 3-fold higher in choroid plexuses isolated from newborn rat compared to adult rat (Angeletti et al., 1998). Western blot and immunohistochemical localization of Slco were also performed in this study, but are difficult to interpret as, at that time, Slco1a5 was mistakenly identified as Slco1a1 in this tissue. As further evidence of organic anion transport maturity at the BCSFB, choroidal epithelial cells isolated from newborn rats efficiently mediate the transport of Slc22 and Slco substrates such as 2,4-dichlorophenoxyacetic acid, benzylpenicillin, zidovudine or taurocholate in an unidirectional CSF-to-blood direction (Strazielle et al., 2003).

#### Developmental localization and expression of SLC15 efflux transporters at the choroid plexus

Among members of the SLC15 family, Slc15a2 is well expressed in the brain, with the highest level in the choroid plexus (Shen et al., 2004). Both mRNA levels and immunohistochemical signals for Slc15a2 were similar in rat choroid plexuses at perinatal and adult stages (Shen et al., 2004; Kratzer et al., 2013). The apical localization of this choroidal transporter suggests its efficacy in exporting neuropeptides, peptide fragments, and peptide-like drugs out of CSF from early on during brain development.

The overall developmental profiles of all ABC and SLC efflux transporters reviewed above are summarized in Table 1. It shows that regarding the expression of efflux transporters, an overall adult-like phenotype is reached earlier at the BCSFB than at the BBB.

**Table 1:** **Comparative analysis of the developmental expression pattern of efflux transporters between the blood-brain- and blood-CSF barriers**.

**Blood-brain barrier**			**Blood-CSF barrier**		
**Major efflux transporters**	**Developmental pattern**		**Major efflux transporters**	**Developmental pattern**	
ABCB1/Abcb1a	↗	mRNA, Prot, Fc	Abcc	=	Fc
ABCG2/Abcg2	=	mRNA, (Prot)	Abcc1	=	mRNA, Prot
Abcc4	↗	mRNA	Abcc4	≤	mRNA
Slc22a8	=	mRNA, Prot	Slc22a8	↗	mRNA
			Slco	↘	Fc
			Slco1a5	=	mRNA
			Pept2/Slc15	=	mRNA (Prot)
**Other multispecific transporters**	**Developmental pattern**		**Other multispecific transporters**	**Developmental pattern**	
Slc22a5	↗	mRNA	Abcb1b	↘	mRNA
Slco1a4	↗	mRNA, Prot	Abcg2	↘	mRNA (Prot)
Slc22a17	=	mRNA	Abcc5	=	mRNA
Slco2b1	=	mRNA	Abcc9	↘	mRNA
Slco2a1→	↘	mRNA	Abcc10	=	mRNA
			Slc22a5	=	mRNA
			Slco1a4	=	mRNA

*↗, ↘, = increased, decreased, similar expression in adult as compared to neonatal stages, respectively. mRNA, assessed by qRTPCR or gene array. Prot, assessed by quantitative western blot. (Prot), assessed by immunohistochemical signal intensity. Fc, assessed by functional studies, using specific substrates. For references, see text*.

### Regulation of efflux transporter associated barrier properties during development

Signaling pathways involved in the early specification of brain barrier cells have been identified in the last decade, pointing out a few molecular cues that specifically regulate the developmental expression of efflux transporters. In addition, these transport systems are modulated in disease and by various xenobiotics via mechanisms that may bear developmental specificities.

#### Pathways involved in the developmental regulation of efflux transporters at blood-brain interfaces

The unique phenotype of brain microvessel endothelial cells compared to other endothelia in the body has long ago raised the hypothesis that surrounding neural cells were responsible for inducing BBB characteristics. A series of grafting experiments generated controversial results regarding this theory (reviewed in Bauer and Bauer, 2000). Arguments in favor of a role for adjacent cells, notably astrocytes emerged from many *in vitro* studies. It was thought for example, that the BBB hallmark transporter Abcb1 was under astrocytic regulation on the basis that its expression and functionality, which are partially lost in brain microvessel endothelial cells in culture were reinduced in endothelial cells co-cultured with glial cells. However, because of the temporal pattern of neural cell differentiation and the late apparition of mature astrocytes which in rat and mouse occurs only around birth, these cells obviously cannot impart the inducing signals that trigger BBB formation in neovessels. Astrocytes nonetheless are essential later for further maturation of the BBB (see infra), or for maintenance of its properties as suggested by the link between some astrocyte diseases and BBB dysfunction (Keller, 2013).

A large number of studies have recently been directed toward identifying the molecules, pathways, and cells responsible for CNS specific vascular differentiation. The Wnt/β-catenin canonical pathway has emerged as a prominent signaling mechanism, promoting angiogenesis and concurrently inducing hallmark features of the BBB, such as the expression of the glucose transporter Slc2a1 (reviewed in Liebner et al., 2011). Wnt factors are secreted as early as embryonic day 9 by neural progenitors. The various studies dedicated to this topic did not examine the direct effects of Wnt signaling on the expression of ABC and SLC efflux transporters at the BBB. DNA binding sites for T cell factor/lymphoid enhancer-binding factor TCF/LEF, the transcriptional activating factor downstream of the Wnt/β-catenin pathway, are present in the human ABCB1 gene promoter (Yamada et al., 2000). Functional evidence linking β-catenin accumulation with intestinal expression of the human ABCB1 gene or the mouse Abcb1a gene has been reported in intestinal cells (Scotto, 2003). Regarding the BBB, activation of β-catenin signaling by GSK-3 inhibition in the human brain endothelial cell line hMEC/D3 as well as in rat brain endothelial cells in primary culture similarly increased ABCB1 expression and function (Lim et al., 2008). The expression of ABCC4 and ABCG2 genes was also modulated in the hMEC/D3 cells in response to β-catenin signaling activation or inhibition, suggesting that the early embryonic expression of Abcg2 described in rat and mouse could depend on the same regulatory pathway. Although direct evidence in the embryonic brain is lacking, these data support the idea that the range of genes associated with the BBB phenotype and induced by the canonical Wnt pathway in newly formed cerebral vessels include efflux transporters. Other regulatory mechanisms are expected to add on this triggering mechanism to explain the different profiles observed among efflux transporters, such as between Abcb1 and Abcg2 for example (see supra).

Retinoic acid, a potent morphogen directing the development of the nervous system, has been proposed as another signaling cue contributing to the induction of the specific BBB phenotype at an early stage of angiogenesis. In human fetal brain, radial glial cells express the final enzyme involved in retinoic acid biosynthesis, while endothelial cells express one of the receptors for this signaling factor (Mizee et al., 2013). Treatment of mice from day 10.5 to day 16.5 of gestation with a retinoic acid receptor antagonist led to the downregulation of several BBB distinct properties in embryos, in particular to a decrease in the expression level of Abcg2 gene. One limitation of this result was that RNA levels were measured on the whole brain including a priori the choroid plexus as well as the subventricular zone, both regions of the brain that express the Abcg2 gene at substantial levels during this period. It is possible that the decrease observed in treated embryos on day 16.5 may not reflect changes at the BBB *per se*, but rather in either one or both of these regions (Orford et al., 2009). Immunostaining of Abcg2 in these animals should clarify this point.

In addition to neural progenitors and radial glial cells, pericytes are present in the early phase of cerebral angiogenesis, and are recruited by the nascent vessels invading the neuroectoderm. A major role of pericytes in influencing the endothelial phenotype has been clearly demonstrated *in vivo* for a number of features, including the loss of transcytotic vesicles, or the induction of the glucose transporter Slc2a1 (Armulik et al., 2010; Daneman et al., 2010b). By contrast, transcript levels for Abcb1a and Abcg2 in endothelial cells of 18-day-old mouse embryos did not differ between pericyte deficient Pdgfrb-/- animals and littermate controls (Daneman et al., 2010b), suggesting that initial induction of these efflux transporters is not under pericyte control.

Because similar deletion of astrocytes *in vivo* is not possible, current evidence for the influence of these cells on the differentiated status of BBB endothelial cells has been gathered using culture systems. One bias in these *in vitro* studies arises from the fact that endothelial cells are isolated from adult brain, thus with an already mature phenotype, and they downregulate some of their BBB-specific properties. For that reason, astrocytic effects observed *in vitro* may reflect not only maturation processes occurring when these cells appear, but also repair mechanisms triggered under pathological conditions. With regard to efflux transporters, the focus has been mainly on ABCB1. Its protein level and function are increased in endothelial cells cocultured with astrocytes, compared to endothelial cells alone (see for example Fenart et al., 1998; Gaillard et al., 2000). This reinduction occurs in the absence of any direct contact between the two cell populations, inferring the role of astrocytic soluble factors. Some of these astrocyte-derived molecules that act on endothelial receptors to promote BBB-specific properties have been identified (Abbott et al., 2006; Alvarez et al., 2011, for a review). They include sonic hedgehog ligands, angiopoietin 1 which is a ligand for the endothelial Tie2 tyrosine kinase receptor, or TGF-β1. Their role on the expression and function of ABC and SLC efflux transporters still need to be evaluated *in vivo*, using animals deficient for these pathways or pharmacological inhibition approaches. Evidence for the role of these signaling molecules in regulating the expression and activity of efflux transporters has been provided in other cell types. For example, activation of the sonic hedgehog pathway associates with elevated therapeutic resistance in cancer cells, in part due to increased protein level and activity of both ABCB1 and ABCG2 (Sims-Mourtada et al., 2007). Consistently, the promoter region of the ABCG2 gene contains a binding site for the downstream transcription factor Gli and is a direct target of sonic hedgehog signaling (Singh et al., 2011). Tie2 activation also promotes a chemoresistance phenotype in gliomas by inducing ABCB1 expression level and activity (Martin et al., 2009), and TGF-β1-mediated increase in Abcb1 expression and activity has been demonstrated in guinea pig brain endothelial cells in culture (Baello et al., 2014).

If significant progress has been achieved in the understanding of how the BBB gains its distinct phenotype, the signaling pathways responsible for the early specification of the choroidal epithelium vs. ependyma remain obscure, in particular concerning the regulation of expression of Slc and Abcc genes. The hepatic nuclear factor 4α (HNF-4α) is a key regulator of differentiation in the liver or the intestine, where it stimulates in particular the transcription of drug metabolizing enzymes and efflux transporters. HNF-4α is expressed in choroid plexus epithelial cells in rat and human at the adult stage, and can bind to regulatory sites in the ABCC1 gene promoter (Niehof and Borlak, 2009). Further work is needed to confirm its potential role in controlling the early choroidal-specific transcription of Abcc1.

#### Modulation of efflux transporters in response to injury and environmental insults during development

The pathogenesis of perinatal injuries most frequently involves hypoxia/ischemia, maternal-fetal infection or inflammation (Aden et al., 2010). These insults are associated with the secretion of proinflammatory cytokines, as well as oxidative/electrophilic stress for which the neonatal brain shows a selectively high susceptibility (Ferriero, 2001). Neuroprotective genes including anti-oxidant genes and efflux transporter genes of the ABC and SLC families are known to be regulated by these stress signals as part of the coordinated defense mechanisms implemented by the cells in response to oxidative stress. The regulatory pathways involve transcription factors that generally act as sensors in the cytoplasm and translocate to the nucleus to increase the transcription of target genes. Oxidative species and the lack of oxygen can be sensed by the nuclear factor erythroid 2-related factor 2 (Nrf2) and the hypoxia inducible factor 1alpha HIF-1α, respectively. There is mounting evidence that these stress sensing factors are expressed and functional in cells of the blood-brain-interfaces. Nrf2-dependent signaling increases Abcb1, Abcg2, and Abcc2 expression levels in rat brain microvessels, and accordingly decreases brain uptake of verapamil, a substrate of Abcb1 (Wang et al., 2014). The efficacy of Nrf2 signaling in improving neuroprotection at the microvasculature during brain development has not been explored. The functionality of the Nrf2-dependent pathway at the BCSFB was assessed *in vitro*, in choroidal epithelial cells, through Nrf2 translocation in the nucleus upon isothiocyanate treatment and transcriptional upregulation of downstream genes (e.g., heme oxygenase, Xiang et al., 2012). It is notable that in rat choroid plexus, Nrf2, HIF-1α, and HIF-1β display a strikingly steady expression from embryonic day 15 to the adult stage (Kratzer et al., 2013). We can therefore speculate that the choroid plexus will react to oxidative/electrophilic and hypoxic stresses at least, by increasing neuroprotective genes at the BCSFB early during development. Yet, this needs further investigation, since hypoxia/ischemia in 9-day-old mice did not change the level of expression of Abcc1 in the choroid plexus, although other target genes for Nrf2, such as heme oxygenase or glutathione-S-transferase a1 were strongly upregulated (D'Angelo et al., 2013). This suggests that Nrf2 signaling is functional in the choroid plexus of young animals, but that not all target genes are equally sensitive at that stage, or share the same kinetic of induction.

Regarding the consequences of inflammatory challenges on efflux transporters, Abcb1 regulation has been investigated in details in freshly isolated rat capillaries (reviewed in Hartz and Bauer, 2010). Its expression and functional activity in adult is differently modulated by short term and long term exposure to pro-inflammatory stimulus. The extent and direction of change in ABCB1 expression is also disease-specific (reviewed in Stolp et al., 2013). During development, inflammation-associated changes in BBB efflux transporters can be stage-dependent. For instance, Abcb1 and Abcg2 efflux function at the BBB increase in response to endothelin-1 in adult, but not in juvenile (21-day-old) rats. This difference may be related to the lower cytokinic response of the BBB to endothelin-1 treatment in juvenile compared to adult animals (Harati et al., 2012b). More work is needed to delineate the role of inflammation in the regulation of efflux transporters at the BBB during development, and the actual window of susceptibility. The choroid plexus is very sensitive to inflammatory challenges, rapidly responding through increased secretion of inflammatory mediators including chemoattractants and pro-inflammatory cytokines. Exposure of the choroidal epithelium *in vitro* to activated immune cells or cytokines leads to a decrease in CSF-to-blood efflux of prostaglandin E_2_, a transport process which is likely mediated by SLC and ABC efflux transporters (Khuth et al., 2005; Batisson et al., 2006). Little is known so far on the regulation of efflux transporters *in vivo* at the BCSFB in the context of perinatal inflammation. Changes in such transport mechanisms that set the CSF levels of different biologically active endogenous organic anions including prostanoids may have dramatic consequences for brain development and maturation, and deserves further exploration.

In different peripheral organs such as the liver, exposure to environmental toxicants or food components can activate additional transcription factors belonging to the nuclear factor family, which in turn trigger the induction of protective genes including efflux transporter genes. Among these xenosensors, the aryl hydrocarbon receptor (AhR), the constitutive androstane receptor (CAR), or the pregnane X receptor (PXR) have efflux transporters among their target genes (see Scotto, 2003 for a review of the mechanisms involved). Current knowledge about these nuclear receptors indicates that they are expressed in cells of the blood-brain interfaces in animals and human. AhR is expressed in human brain microvessels, while additional expression of PXR and CAR genes has been reported for the rodent BBB (Dauchy et al., 2008; Hartz and Bauer, 2010). Upon ligand activation, these nuclear factors were shown to induce the expression and/or function of Abcb1, Abcc2, or Abcg2 (reviewed in Hartz and Bauer, 2010). It is still unknown whether nuclear receptors are expressed and modulate efflux transporters at the BBB during development.

In choroid plexus, gene expression can be detected for a wide range of these transcription factors in prenatal, postnatal, and adult stages of rat development, and their role in the coordinated induction of detoxifying enzymes and transporters at the BCSFB has been suggested (Kratzer et al., 2013).

## Conclusion

The current limited data available in the literature reveal transporter-dependent developmental profiles of expression in blood-brain interfaces both in laboratory animals and in human. The developmental pattern of a given transporter can also differ between the BBB and the BCSFB (Table 1). The BCSFB achieves an “adult-like” phenotype earlier than the BBB. For instance, in the latter, Abcb1 displays a major increase in expression postnatally. On the basis of recent studies on early BBB differentiation, we can speculate that neural progenitors and radial glial cells have a major role in triggering the endothelial expression of certain efflux transporters via Wnt and retinoic acid signaling. This would provide some degree of protection against deleterious compounds to the developing brain. Once vascular networks have formed, newly generated astrocytes induce further differentiation of the BBB, through the sonic hedgehog pathway. The concept of barrier immaturity during brain development may therefore hold for selected efflux transporters at the BBB. This novel view of a restricted barrier capacity of the developing brain endothelium is independent from the efficacy of its tight junctions sealing the paracellular pathway, which is established early during prenatal development. It needs to be taken into account when neonatal and pediatric treatments are discussed. The developmental regulation of efflux transporters at the choroid plexus remains obscure. Further work is needed on the signaling cues responsible for specifying the choroidal phenotype of the neuroepithelium and in particular for governing the expression of efflux transporter genes.

A number of choroidal ABC transporters, as well as choroidal and endothelial SLCO transporters display a higher expression or activity in developing animals than in adults. Additional studies, tackling especially the functionality and substrate specificity of these transporters, are necessary to better appreciate the significance of their stage-specific expression.

In the context of developmental diseases, aberrations in one of the signaling pathways that induce efflux transporter-dependent protective functions in blood-brain interfaces may lead to substantial changes in fetal/neonatal brain exposure to undesired substrates, and subsequently alter normal brain development.

Choroid plexus studies suggest that from early on during development, brain efflux may be enhanced following exposure to xenobiotic inducers including drugs and environmental toxins. The modulation of efflux transporter expression and function by oxidative stress and inflammation often associated with perinatal injuries deserves further investigation. A better understanding of the regulatory pathways, in particular the relative contribution of the different transcription factors sensing oxidative stress or xenobiotics during development, is clinically relevant. It may open new opportunities for therapeutic manipulation designed to improve or restore blood-brain interface neuroprotective functions in the context of perinatal injuries.

### Conflict of interest statement

The authors declare that the research was conducted in the absence of any commercial or financial relationships that could be construed as a potential conflict of interest.

## Footnotes

^1^ Capitalized characters are used when referring to human transporters or to transporters listed in a general context. Minor characters are used when specifically referring to rodent transporters.

## Abbreviations

BBB: blood-brain barrier
BCSFB: blood-CSF barrier
ABC: ATP-Binding Cassette
OATP: organic anion transport polypeptide
HNF-4 α: hepatic nuclear factor 4α
Nrf2: nuclear factor erythroid 2-related factor 2
AhR: aryl hydrocarbon receptor
CAR: constitutive androstane receptor
PXR: pregnane X receptor.
